# An Action Research Method for Generating Human-Centered COVID-19 Caregiving Interventions

**DOI:** 10.1093/geroni/igaa057.3423

**Published:** 2020-12-16

**Authors:** Natalie Leonard, Alicia Carmichael, Jeannette Jackson, Linde Huang, Aalap Doshi, Amanda Leggett, Sheria Robinson-Lane, Richard Gonzalez

**Affiliations:** 1 University of Michigan, Ann Arbor, Michigan, United States; 2 The University of Michigan, Ypsilanti, Michigan, United States

## Abstract

Caregiving for post-intensive care COVID-19 patients is an important determinant of successful recovery, including the reduced likelihood of ICU readmission. With possible ICU readmissions coinciding with a second wave of the pandemic, researchers and clinicians at the University of Michigan sought to develop a patient and caregiver-informed intervention that was remote, accessible, and could be immediately delivered. The resulting study, Health Enhanced by Adjusting and Recovering Together, reinforces these imperatives common in action research frameworks. Action research, emerging itself from a tumultuous time (1930s-40s) paralleling the COVID-19 pandemic, is a pragmatic research approach with the explicit goal of resolving a community problem or enacting social change--and doing so quickly. Here, we demonstrate a unique method for rapid intervention development that intertwines elements of (a) Human-centered design, for the purpose of a people-focused outlook, (b) Action research, for the purpose of rapid intervention, and (c) Traditional qualitative analysis, for the purpose of knowledge creation. The result of this combined method is an efficiently developed intervention that, while imperfect, is a user-centered, contextually-relevant viable product that can be quickly disseminated, tested, and further refined. The method presented is timely and relevant to other clinical and research teams addressing caregiving during the COVID-19 pandemic.

